# Lea Ferreira Camillo-Coura (★1932 †2023) Always first

**DOI:** 10.1590/0037-8682-0107-2023

**Published:** 2023-04-14

**Authors:** Cláudio Tadeu Daniel-Ribeiro, Octavio Fernandes da Silva

**Affiliations:** 1 Instituto Oswaldo Cruz, Centro de Pesquisa, Diagnóstico e Treinamento em Malária, Laboratório de Pesquisa em Malária, Rio de Janeiro, RJ, Brasil.; 2 Ministério da Saúde, Secretaria de Vigilância em Saúde e Ambiente, Brasília, DF, Brasil.; 3 Academia Nacional de Medicina, Rio de Janeiro, RJ, Brasil.; 4 Labi Exames, Rio de Janeiro, RJ, Brasil.; 5 Academia de Medicina do Rio de Janeiro, Rio de Janeiro, RJ, Brasil.

Professor Lea Ferreira Camillo-Coura was born on June 25, 1932 and died on January 28, 2023 in Rio de Janeiro, RJ. Her career is filled with “first times” and “first placements”. Lea, the daughter of the naval officer Benedicto Barbosa Camillo and Universina Ferreira Barbosa Camillo, graduated in Medicine from the *Universidade Federal do Rio de Janeiro (UFRJ)*, then called the *Faculdade Nacional de Medicina* of the *Universidade do Brasil*, first in her class, in 1957.

In 1963, she undertook postgraduate studies at master's level at the London School of Hygiene and Tropical Medicine, University of London, thanks to a scholarship from *Fundação da Casa do Brasil* in UK. Back in Brazil, she developed activities at the Clinic for Infectious and Parasitic Diseases at the *Hospital São Francisco de Assis, UFRJ*, from 1964 to 1971, where, after being approved in a public contest proving her knowledge in Clinical Infectious and Parasitic Diseases, and complying with legal requirements, she was promoted through titles analysis to the position of Associate Professor.

Lea Camillo-Coura participated in several symposia and served as *ad hoc* consultant for the Ministry of Health, in Brazil, and as a temporary consultant for the WHO, in different countries around the world, on matters related to Intestinal Parasites, a subject of study to which she dedicated her entire career. She became Professor of the Discipline of Epidemiology (1973) and Teaching Coordinator of the Department of Preventive Medicine of the Faculty of Medicine, UFRJ (1974-1978). She then took over the Discipline of Infectious and Parasitic Diseases as Full Professor (1975-1988) at the *Fundação Técnico Educacional Souza Marques* and became Head of the Community Action Service at the University Hospital of UFRJ (1977-1982).

A Full Professor of both the Graduate Course in Infectious and Parasitic Diseases at the *Instituto de Pós-Graduação Médica Carlos Chagas* (1980-1987) and the Postgraduate Course in Infectious and Parasitic Diseases, as well as a Deputy Director for Postgraduate courses from the Faculty of Medicine at *UFRJ* (1982), Lea created the Professional Psychopedagogical Guidance Program (*POPPE*), in 1982. She was also Head of the Department of Tropical Medicine at the *Instituto Oswaldo Cruz (IOC, Fiocruz)*, in 1981 and 1982, which her husband JR Coura would eventually head up until the end of the Research Departments at the IOC.

**Lea Camillo-Coura:**
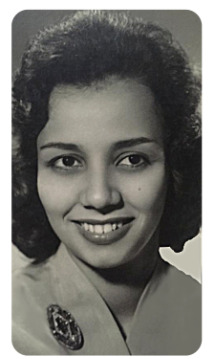
at the Pavilhão Carlos Chagas (São Francisco de Assis Hospital, 1960s)

For 14 years, Lea has been the Scientific Editor of the *Revista da Sociedade Brasileira de Medicina Tropical (RSBMT)*, a journal created in 1967 by Lea’s and JR Coura’s professor, José Rodrigues da Silva.

Upon leaving the editorial board in 1982 and handing it over to Professor Aluízio Prata, Lea recalled the difficulties faced in meeting the journal’s expenses with the help of some pharmaceutical laboratories and the annuities paid irregularly by the Members of the Society^1^. Possibly because of having embraced and protected the journal so fiercely for so many years, Lea concluded that “maintaining this Journal... was to keep José Rodrigues da Silva’s dream alive, and it meant to me the greatest proof of dedication and love for SBMT, which which I am proud to have founded and where I am proud to belong.” 

As member of the Research Ethics Committee (CEP) of *Fiocruz* (1990) and Coordinator of the CEP of the *Instituto Nacional de Infectologia (INI) Evandro Chagas, Fiocruz*, for many years, Lea has shown her ethics, uprightness of character and dignity, always performing her duties in an entirely impartial manner. She supervised master’s and PhD students in the Tropical Medicine and Parasite Biology courses at the *Instituto Oswaldo Cruz*, Public Health at the *Escola Nacional de Saúde Pública (ENSP)* and Clinical Research in Infectious Diseases at *INI, Fiocruz*.

Lea Camillo-Coura became Emeritus Researcher at the *CNPq* (2005) and received the Gerhard Domagk Award (1970); the Naval Merit Medal (Tamandaré), the Santos Dumont Merit (1982); the Peacemaker Medal (1985) and the Honorary Citizenship of the Municipality of Rio de Janeiro. Elected Woman of the Year (Medicine) by the National Council of Women of Brazil (1991), she received from Fiocruz the Hortência Hurpia de Hollanda Medal, for her work in favor of education and, from the Presidency of the Republic, the grade of *Comendador da Ordem do Mérito Científico do Brasil*.

First woman to be appointed Honorary Member of the *Academia Brasileira de Medicina Militar*, later becoming Full and Emeritus Member, she was founder of the *Sociedade Brasileira de Medicina Tropical*, as well as of the *Academia de Medicina do Rio de Janeiro*, and became an Honorary Member of the *Academia Brasileira de Medicina e Reabilitação*.

Lea left three children: Lucia, Luciana and Evandro, and three grandchildren: Leonardo and Guilherme, children of Evandro, and Beatriz, Luciana’s daughter. Lucia and Luciana described Lea as “an extremely intelligent woman, having thousands of distinct aptitudes. A meticulous pianist (who didn't like to commit mistakes, even though she didn't use to practice that much), she prepared her whole family’s income tax returns annually and took care of ferns, which she particularly appreciated. Lea was a sovereign and lofty person who used to interact with people from all cultures and all social classes, talk about any topic and help everyone, in any way she could...”

Lea liked to receive friends and students at her residence in Urca to the sound of classical music, when she used to show them affectionately each of the Camillo family’s relics or the souvenirs collected on business trips after she got married.

One of, if not the most impressive achievements in Lea Camillo-Coura’s career should be left to the end: she became the first woman to be elected Full Member of the *Academia Nacional de Medicina (ANM)*, 114 years after Mme. Marie Josephine Mathilde Durocher, the first female Full Member of the *ANM* (appointed at the request of Emperor Pedro II). Since 1985, Lea has occupied the chair 82, patronymic of Antonio Dias de Barros, having as predecessor the Academician Annibal (Rocha) Nogueira (Júnior), as Patron the Academician Deolindo (de Souza Gomes) Couto and as successor the Academician Carlos Alberto Mandarim-de-Lacerda^2^. Lea went on to become Emeritus at *ANM* (2010) before also becoming Emeritus Researcher at *Fiocruz* (2014), when she concluded her speech declaring with conviction: “I have fought the good fight, finished the race, kept the faith...”

You did it, dear Lea!
